# Time orientation and health-related behaviour: Measurement in general population samples

**DOI:** 10.1080/08870440701813030

**Published:** 2009-03-04

**Authors:** Rachel A. Crockett, John Weinman, Matthew Hankins, Theresa Marteau

**Affiliations:** Department of Psychology, King's College London, London, UK

**Keywords:** time orientation, health-related behaviour, diabetes screening, socioeconomic status

## Abstract

Research on health behaviour and time orientation has been hindered by a lack of consensus about appropriate measurement. Study 1 assessed the reliability of the Consideration of Future Consequences Scale (CFC) and the Zimbardo Time Perspective Inventory (ZTPI) in a general population sample (*n* = 300). Although more reliable, the CFC was less readable. Study 2 assessed the validity of a shortened ZTPI, measuring future and present orientation, and the full CFC. The measures had good discrimination to distinguish interpersonal differences. Construct validity of present, but not future, orientation as measured by the ZTPI, was evidenced by its mediation of the association between socioeconomic status and expectations of participating in diabetes screening. The CFC mediated this relationship more weakly. Further investigation of present orientation in understanding health-related behaviour is warranted.

Health behaviour is characterised by immediate effort for possible future gain. Given this, one psychological variable that might add power to models of health behaviour is time orientation. Time orientation suggests that people use information about the timeframe in which an event occurs to evaluate and respond to the event. People tend to be motivated more either by future or by present goals in making decisions, reflecting greater future or present time orientation (Simons, Vansteenkiste, Lens, & Lacante, 2004). Such tendencies can be stable characteristics that may develop in response to socialisation or life events (Stein, Sarbin, & Kulik, 1968; Wills, Sandy, & Yaeger, 2001). This does not preclude the possibility that the extent to which future or present orientation motivates decision making may vary across different situations, although no research has been identified which addresses this possibility.

Future orientation has been associated with a clear vision of the future and awareness of the effects of current actions on future outcomes (Rothspan & Read, 1996). Studies report associations between future orientation and activities involving immediate effort for future gain including education (Brown & Jones, 2004; D'Alessio, Guarino, De Pascalis, & Zimbardo, 2003), practicing safe sex (Rothspan & Read, 1996), and engaging in physical activity and healthy eating (Luszczynska, Gibbons, Piko, & Tekozel, 2004). Present orientation is associated with a limited sense of control, fatalism and preference for behaviours with immediate rewards (Rothspan & Read, 1996; Zimbardo & Boyd, 1999). It has been associated with risky sexual behaviour (Rothspan & Read, 1996), substance abuse (Keough, Zimbardo, & Boyd, 1999) and risky driving (Zimbardo, Keough, & Boyd, 1997). However, present orientation is not necessarily related to poorer well-being. Some research has indicated that in situations requiring immediate action, such as homelessness, greater present orientation, may be adaptive (Epel, Bandura, & Zimbardo, 1999). In relation to Type 1 diabetes adaptation is predicted by both future- and present-orientation pleasurable feelings. The concurrent presence of both future and present orientation indicates that these are not simply the reverse poles of one construct, a suggestion given further support by evidence that the two measures each account for unique variance in the study of risky behaviour (Keough et al., 1999; Zimbardo et al., 1997).

Future orientation has been shown to contribute to the modelling of decisions about engaging in health promoting activities. For example, responses to persuasive communications to attend health screening where either the benefits of participation were presented as immediate and the harms as future, or the benefits as future and the harms as immediate, were patterned by future orientation (Orbell & Hagger, 2006; Orbell, Perugini, & Rakow, 2004).

Furthering the understanding of decisions about participation in health-related behaviour depends on greater understanding of the socioeconomic context in which those decisions are made. Those of low socioeconomic status (SES) are less likely than those of high SES to participate in health-enhancing behaviours (Fernandez et al., 2006; Kim, Thompson, Marteau, & Scott, 2004; Wardle & Steptoe, 2003). While lack of material resources, such as money to pay for gym membership, contributes to poorer health behaviour, it is not a complete account (Adler et al., 1994; Lantz, Weigers, & House, 1997). Low participation by these groups is a matter of concern. Those who are more deprived have poorer health, are more likely to suffer from chronic illnesses such as diabetes (Congdon, 2006) and experience severe adverse effects (Bachmann et al., 2003). Understanding the psychological constructs that are associated with both social deprivation and participation in health-related behaviours should therefore be a key concern in the development of models of health decision making. There is some indication that time orientation is related to both screening uptake (Orbell et al., 2004; Orbell & Hagger, 2006) and SES (D'Alessio et al., 2003; Nurmi, 1987): thus time orientation might add explanatory power to models of health decision making. In order to fully identify the possible role of time orientation in such decisions, key issues remain to be fully addressed: first the most appropriate measure of time orientation and second whether current research, conducted largely in student samples, can be applied to the general population. Of the 17 papers reporting empirical studies of time orientation cited in this article, 11 used school and college students. As future orientation has been associated with education (Brown & Jones, 2004; D'Alessio et al., 2003) and age (D'Alessio et al., 2003) it is unlikely that the time orientation of school and college students reflects that of general populations, nor that the associations found in student samples will reflect patterns of association in general population. For example, there is some suggestion that while present orientation may be associated with self-efficacy in an adult population (Epel et al., 1999), the associations are very low in a student population (Lennings, 2000).

The aims of the two studies reported in this article are to explore the reliability of two measures of time orientation in a general population sample and to explore the validity of time orientation as an explanatory variable in models of health-related behaviour. These aims will be addressed within the context of a topical health behaviour, that is, participation in diabetes screening which is currently under review for introduction as a national screening programme in England (www.nsc.nhs.uk).

## Study 1: A comparison of the reliability and readability of two measures of time orientation

The two most widely used measures of time orientation are the Consideration of Future Consequences Scale (CFC) (Strathman, Gleicher, Boninger, & Edwards, 1994) and the Zimbardo Time Perspective Inventory (ZTPI) (Zimbardo & Boyd, 1999). Strathman and his colleagues describe the concept the CFC measures as being one aspect of future orientation, that is, ‘thought about future consequences of current actions’. They report the evolution of the CFC's 12 items in a series of studies within a student population. When factor analysed a single factor accounting for 94.6% of the variance was found. Validity was illustrated through association with measures, such as delay of gratification, with which the CFC conceptually would be expected to share a relationship, and by showing that greater CFC was predictive of concern for health and for the environment (Strathman et al., 1994). The ZTPI has been known as both the Stanford Time Perspective Inventory and latterly as the Zimbardo Time Perspective Inventory. The measure has had various forms with different items and numbers of items measuring different combinations of five factors: ‘future’; ‘present hedonistic’; ‘present fatalistic’; ‘past negative’ and ‘past positive’. The variance explained by different versions of the ZTPI is typically low (29%: two factors, Epel et al., 1999; 40%: four factors, Klingemann, 2001; 31%: four factors, Lennings, 2000 and 36%: five factors, Zimbardo & Boyd, 1999) although in one study the variance explained by a three-factor model did reach 65% (D'Alessio et al., 2003).

An assessment of the reliability of both measures in one general population sample would provide robust comparative evidence of their measurement properties. The first aim of the current study is to compare the reliability of the two measures by examining their factor structure, internal consistency and test-retest reliability. As a superficial examination of the CFC suggests it has low readability the second aim is to assess and compare the readability of the CFC and ZTPI.

## Method

### Design

#### A questionnaire-based descriptive survey

*Participants*. The sample of 300 adults was structured to reflect the English population in terms of age and gender with a third being drawn from each of the North, South and Midlands of England. To ensure a range of literacy levels, half of the samples were selected to have no education beyond compulsory schooling. Sample size was based on the number needed to conduct Principal Components Analysis (PCA). Although a sample of 150 is sufficient where factors include some items with loadings > 0.80, where factor loadings are lower, as in the case of the ZTPI (D'Alessio et al., 2003; Epel et al., 1999; Klingemann, 2001), a sample of 300 is required (Tabachnick & Fidell, 2001).

*Procedure*. Home-based interviews were conducted by a research agency. Participants were given information about diabetes screening (Appendix 1) and questionnaires were completed by interviewers on behalf of participants. About 6–8 weeks after initial data collection, 43 participants were re-interviewed to collect follow-up data on the CFC and ZTPI.

### Measures

#### Time orientation

1. The 12-item CFC (Strathman et al., 1994) measuring thought about future consequences of current actions (Appendix 2). Each item is measured on a five-point scale from 1 (very uncharacteristic of me) to 5 (very characteristic of me).
2. The 22-item version of the ZTPI (D'Alessio et al., 2003; Keough et al., 1999; Zimbardo et al., 1997) assessing time orientation (Appendix 3). Each item is measured on a five-point scale from 1 (very uncharacteristic of me) to 5 (very characteristic of me).

*Demographic data*. Gender; age assessed by asking participants to indicate their age by selecting one of six age bands; ethnicity assessed by asking participants to indicate 1 of 10 possible descriptions of their ethnic origin.

### Analyses

Readability of the measures was assessed using the internet resource ‘Readability Statistics’ (http://streamer.rit.edu/~jeffs/services/TestReadability.html). This calculates the Gunning Fogg Index (GFI) giving the number of years of education needed to understand a text, with a recommended score between 8 and 10. PCA with varimax rotation using SPSS version 12 was used to explore the factor structure of the two measures. Further analysis of the CFC by structural equation modelling (SEM) was undertaken using AMOS 4.0. Reliability of factors was assessed using Cronbach's α. Test-retest reliability was assessed using Pearson's correlation coefficient (*r*) between factor scores at the two time points. *Post hoc* analyses explored the impact of readability on the reliability of the CFC. Reliability might be artificially inflated if participants who did not understand an item responded at the mid-point of the scale to indicate neither agreement nor disagreement. To assess this, items from the CFC and ZTPI were divided into four groups from the most to the least readable and the mean percentage responding at the mid-point for each group was plotted.

## Results

About 49% of the sample was male, 12% were from minority ethnic groups, 62% were aged between 18 and 49 years old and 38% were aged over 50 years. About 43% did not have education beyond the minimum school leaving age (currently 16 years).

### Readability analyses

While the ZTPI had good readability with a mean of 7.62 (SD: 3.55), that of the CFC was significantly poorer [*t*(32) = 5.54, *p* < 0.001] with a mean 14.49 (SD: 3.26). Only one CFC item had readability at the recommended level, while three items had readability above 15 and one of these had a score of 23.6 (Appendices 2 and 3).

### PCA and SEM

An initial solution of two factors with eigenvalues > 1, together explaining 51.5% of the variance, was indicated by PCA of the CFC and confirmed by examination of the scree plot. Rotation of the solution indicated that seven items loaded on the first factor, with loadings between 0.57 and 0.80, explaining 29.36% of the variance. Five items loaded on the second factor, with loadings between 0.56 and 0.73, explaining 22.15% of the variance (Table 1).

**Table 1:** Factor structure and loadings after principle components analysis and varimax rotation of the CFC and the ZTPI with means and SDs for each item.

CFC Item	Description	Factor loading	M (SD)
Factor 1: reversed items			
11	Satisfy immediate concerns and take care of future later	0.80	3.14 (1.07)
4	Behaviour influenced by immediate outcomes	0.76	3.16 (1.11)
3	Satisfy immediate concerns & future take care of itself	0.73	3.11 (1.17)
10	Sacrificing now unnecessary	0.73	3.19 (1.12)
9	Ignore problem-warnings because will be resolved before crisis	0.64	3.52 (1.16)
5	Convenience is big factor in decision making	0.61	2.66 (0.99)
12	Specific outcomes more important than distant outcomes	0.57	2.84 (0.94)
	Variance = 29.36	Eigenvalue = 4.35	α = 0.84
Factor 2: non-reversed items			
1	Consider things in future and influence daily behaviour	0.73	3.53 (0.93)
8	Distant consequences more important than immediate consequences	0.72	3.47 (0.82)
7	Take warnings about negative outcomes seriously	0.71	3.83 (0.88)
2	Engage in behaviour to achieve outcomes many years later	0.67	3.21 (1.01)
6	Willing to sacrifice immediate happiness	0.56	3.11 (1.13)
	Variance = 22.15	Eigenvalue = 1.34	α = 0.73
ZTPI Future orientation			
5	Future plans well laid out	0.75	3.93 (1.41)
2	Thinking about future is pleasant	0.66	3.72 (1.07)
3	Set goals and specific means to reach them	0.63	3.83 (0.98)
4	Meeting tomorrow's deadline comes before tonight's play	0.48	3.41 (1.15)
	Variance = 11.06	Eigenvalue = 4.08	α = 0.66
Hedonism			
21	Get drunk at parties	0.80	2.36 (1.50)
22	Take risks to put excitement in my life	0.76	2.26 (1.31)
18	Fun to gamble when I have extra money	0.52	1.93 (1.22)
15	Getting together with friends is one of lifes important pleasures	0.49	4.28 (0.86)
14	Do things impulsively	0.46	3.07 (1.25)
	Variance = 9.94	Eigenvalue = 2.23	α = 0.61
Conscientiousness			
12	Keep working at difficult uninteresting task to get ahead	0.77	3.50 (1.09)
10	Complete projects on time	0.62	3.86 (0.91)
7	Upsetting to be late for appointments	0.57	4.06 (1.14)
13	Resist temptations when there is work to be done	0.53	3.62 (1.06)
	Variance = 9.85	Eigenvalue = 1.88	α = 0.66
Present orientation			
20	Do not do things good for me if they do not feel good now	0.77	3.26 (1.13)
19	More important to enjoy what you are doing than to get done on time	0.7	3.30 (1.07)
17	Live one day at a time	0.59	3.51 (1.17)
	Variance = 9.16	Eigenvalue = 1.59	α = 0.59
Items which loaded to factors not included in the final solution.			
1	Persons day planned ahead each morning		
6	Useless to plan too far ahead^a^		
8	Tend to lose my temper		
9	Irritated when people keep me waiting		
11	Make lists of things to do		
16	If don't get done on time, don't worry^b^		

a Also loaded.−0.45 on future factor.

b Also loaded 0.46 on present factor.

Examination of the rotated component matrix indicated that Factor 1 contained all the reverse-scored items and no other items. It was therefore hypothesised that the two-factor solution was an artefact of the item wording. Competing models were explored using SEM.

Three models for the CFC were specified and compared. Model 1 modelled the single-factor solution initially expected, that is, one construct (future orientation) measured by 12 indicators (the questionnaire items). Model 2 modelled the two-factor model indicated by PCA, that is, two correlated constructs measured by five indicators (questionnaire items 1,2,6,7 and 8) and seven indicators (items 3,4,5,9,10,11 and 12), respectively. Model 3 replicated Model 1 but added paths between the error terms on the reverse-scored items (items 3,4,5,9,10,11 and 12) to allow for systematic error variance due to processing bias of the reverse-worded items. The goodness-of-fit statistics indicated that the single-factor solution, Model 1, was not consistent with the data: χ^2^(54) = 394.6, *p* < 0.001; GFI = 0.79; Normed fit index (NFI) = 0.69; Root mean square error of approximation (RMSEA) = 0.15 (95%CI: 0.13–0.16). Model 2 showed a better fit, though again, the fit was not adequate: χ^2^(53) = 214.1, *p* < 0.001; GFI = 0.89; NFI = 0.83; RMSEA = 0.10 (95%CI: 0.09–0.12). Only for Model 3 were the goodness-of-fit statistics satisfactory: χ^2^(33) = 86.1, *p* < 0.001; GFI = 0.95; NFI = 0.93; RMSEA = 0.07 (95%CI: 0.06–0.09). The results thus supported the hypothesis that the two-factor solution is an artefact of the questionnaire wording and that the CFC measures one construct, with systematic bias in responses due to the reversal of the sense of items 3,4,5,9,10,11 and 12. Consistent with this, the single factor had a Cronbach's α of 0.83 and a test-retest correlation coefficient of 0.80.

PCA of the ZTPI indicated a solution of six factors with eigenvalues > 1. Examination of the scree plot suggested that four factors explaining 44.5% of the variance should be retained. The first factor of the rotated solution, ‘future orientation’, contained four items with loadings between 0.48 and 0.75 explaining 11.06% of the variance and having a Cronbach's α of 0.66 and a test-retest correlation coefficient of 0.71. The second factor contained five items with factor loadings between 0.46 and 0.80, explaining 9.94% of the variance and having a Cronbach's α of 0.61 and a test-retest correlation coefficient of 0.81. Most of the items concerned enjoyment: only one item, with the lowest loading, was time related. The factor was therefore labelled ‘hedonism’. The third factor contained four items with loadings between 0.53 and 0.77 explaining 9.85% of the variance and having a Cronbach's α of 0.66 and a test-retest correlation coefficient of 0.67. This factor was termed ‘conscientiousness’ because only two of the items had content related to time and conscientiousness was the theme that linked all the items. The final factor, termed ‘present’, contained three items having loadings from 0.59 to 0.77, all indicating an orientation to the present. This explained 9.16% of the variance and had a Cronbach's α of 0.59 and a test-retest correlation coefficient of 0.62. Six items were omitted because their greatest loading was on factors that were not part of the final solution (Table 1).

**Figure 1:**
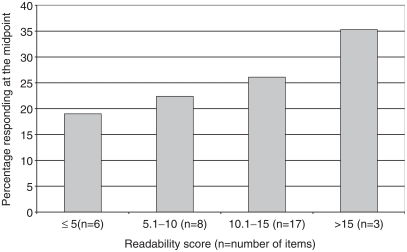
Association between item readability and proportion of participants responding at the scale midpoint.

Associations between the CFC and the four factors of the ZTPI are shown in Table 2 The CFC was significantly positively associated with the ZTPI future and negatively associated with the ZTPI present. ZTPI future was also negatively associated with ZTPI present. Conscientiousness was significantly positively associated with future orientation as measured by both the ZTPI and the CFC and negatively associated with present orientation while the hedonism was negatively associated with both CFC and ZTPI future and positively associated with present orientation. These relationships suggest that conscientiousness and hedonism measure related although different concepts to time orientation. The possible impact of readability on responses is illustrated in Figure 1 plotting item readability against the percentage of participants responding at the mid-point of the scale.

**Table 2:** Associations between the one factor CFC and the four factor of the ZTPI-22 (Spearman's rho correlations).

	ZTPI Future	ZTPI Hedonism	ZTPI Conscientiousness	ZTPI Present
CFC	0.38***	−0.20***	0.35***	−0.40***
ZTPI Future		−0.15**	0.39***	−0.22***
ZTPI Hedonism			−0.14*	0.19***
ZTPI Conscientiousness				−0.23***

Notes:

* *p* < 0.05,

** *p* < 0.01 and

*** *p* < 0.001.

A Spearman's rho correlation indicated that there was a significant association between poor item readability (indicated by a high GFI score) and the percentage frequency of responses at the mid-point of the scale (*r* = 0.43, *n* = 34, *p* < 0.05) suggesting that difficulty understanding the question increased responses at the neutral mid-point of the response scale.

## Discussion

While PCA indicated a two-factor solution for the CFC, further analysis using SEM revealed that this solution was an artefact of the item wording. A four-factor solution was indicated for the ZTPI, although only two of these factors explicitly reflected time orientation. Scoring the CFC as a one-factor model gave a measure with greater reliability than the two time-related factors of the ZTPI. However, the CFC had much poorer readability than the ZTPI. Further examination of item responses indicated that as readability decreased the mid-point of the response scale was increasingly used (indicating neither agreement nor disagreement).

As the CFC had more difficult items to which more respondents answered at the neutral mid-point, part of the apparent reliability of the CFC might be a consequence of the way that people respond to items that they do not understand. In relation to the ZTPI this study suggests that the inconsistent factor structures found in prior research are likely to be the result of the inclusion of items that do not specifically measure time orientation. However, it is possible that had the longer 56-item ZTPI been used, all the factors emerging would have been more clearly time orientated. While internal reliabilities for the future and present scales were not high, they are comparable to those found in previous studies. In six previously reported studies the reliability of the ZTPI future orientation factor ranges from 0.58 to 0.84, with an average of 0.72 and reliability of present orientation ranges from 0.47 to 0.79 with an average over these studies of 0.64 (Epel et al., 1999; Keough et al., 1999; Rothspan & Read, 1996; Wills et al., 2001; Zimbardo et al., 1997; Zimbardo & Boyd, 1999). Test-retest reliabilities for the future and present factors were slightly lower in the current study in comparison to those found in previous studies (0.73 for the future scale and 0.70 for the present scale Zimbardo et al., 1997). These results suggest that while the CFC is a more reliable measure, the ZTPI is significantly more readable. A comparison of the validity of the two measures would provide further evidence as to the properties of the two measures.

## Study 2: Comparing the validity of two measures of time orientation for use in health behaviour research

Validations of both the CFC (Strathman et al., 1994) and ZTPI (Zimbardo & Boyd, 1999) have been conducted in student samples, but it is important to validate the measures in general population samples not only because student samples do not represent the general population in terms of either literacy or age but also because if time orientation is to be identified as a variable that contributes to explaining health-related behaviour the variable should be validated within general population samples.

Validity can be assessed by examining the presence of associations between concepts or outcomes that are expected to be related, and the absence of associations between concepts and outcomes that are expected to be unrelated. Additionally, the discrimination of the questionnaire can be assessed, that is, whether the questionnaire is capable of distinguishing between respondents (Ferguson, 1949; Kline, 1993).

Construct validity is the extent to which the results given by a measure reflect the definition of the psychological construct (Kline, 1993). Evidence for the construct validity of time orientation would be found if it were shown that time orientation contributed to understanding an established relationship between variables. It is well established that screening participation is lower in those from lower SES groups than in those from higher SES groups (Kim et al., 2004; McCaffery, Wardle, Nadel, & Atkin, 2002; Sutton & Rutherford, 2005). Health-related behaviours, such as screening participation, require immediate effort for possible future gain and thus variation in uptake of screening may be related to different patterning of time orientation in different groups. As well as research indicating that future orientation is associated with healthy behaviour and present orientation with more risky behaviour (Keough et al., 1999; Orbell et al., 2004; Orbell & Hagger, 2006; Rothspan & Read, 1996; Zimbardo et al., 1997), there is some evidence that future orientation increases as SES increases (Nurmi, 1987; Stein et al., 1968). However, no research has attempted to establish a mediating role for both present and future orientation in predicting health behaviour in general population samples. Identification of a mediating role would contribute to explaining the relationship between SES and screening participation.

The objective of Study 2 was to extend the existing knowledge base relating to time orientation and health behaviour. Specifically, the first aim was to assess the discrimination of the CFC and ZTPI by calculating coefficient δ_*G*_ (Hankins, 2007). δ_*G*_ is a non-parametric statistic indexing the degree of inter-individual discrimination obtained by the questionnaire. It is derived from Ferguson's coefficient δ (Ferguson, 1949; Kline, 1993) modified for use with Likert-type scales and ranges between 0.0 (no discrimination: all respondents score the same) and 1.0 (maximum discrimination: all scores occur with equal frequency). The value of δ_*G*_ is maximised if the distribution of scores is rectangular, and will typically exceed 0.9 for a normal distribution of scores. It may be straightforwardly interpreted as the ratio of actual discriminations made by the questionnaire to the maximum possible number of discriminations: for example, a δ_*G*_ of 0.75 indicates that only 75% of all possible discriminations were made. The second aim was to assess the construct validity of time orientation in predicting the uptake of a health behaviour, specifically diabetes screening, by testing two hypotheses: (1) future orientation mediates the relationship between higher SES and higher expectations of participation in diabetes screening and (2) present orientation mediates the relationship between lower SES and lower expectations of participation in diabetes screening.

## Method

Study 2 used a sub-set of the data used in Study 1; thus design, participants and procedure were the same as for Study 1.

### Measures

#### Time orientation

Assessed using two measures:

1. CFC: The 12-item version of the CFC (Strathman et al., 1994) described in Study 1.
2. ZTPI-R: A brief nine item form of the ZTPI. Because the results of Study 1 suggested that the measurement of time orientation by the ZTPI was confounded by items actually measuring other constructs, a shortened version was identified. The seven items which loaded on to the future and present scales in the initial factor analysis, along with the two items that were omitted in that analysis but had a loading above 0.4 one on each of these factors, respectively, were entered into a PCA. The initial statistics indicated two factors with eigenvalues > 1, explaining 47.3% of the variance. This solution was confirmed by examination of the scree plot. Rotation of the solution indicated that the five items relating to future orientation had loadings ranging from 0.52 to 0.79 on the first factor which had an eigenvalue of 2.64 and explained 29.26% of the variance. The other four items all related to present time orientation and had loadings ranging from 0.64 to 0.70 on the second factor which had an eigenvalue of 1.63 and explained 18.03% of the variance. The Cronbach's α were 0.67 for the future factor and 0.62 for the present one, indicating some increase in reliability, particularly for the present orientation factor. The test-retest reliability coefficient was 0.66 for the future factor and 0.64 for the present factor.

*Expectations of participation in screening*. The mean score of three items (Cronbach's α 0.85) based on those used in similar studies (Orbell et al., 2004; Orbell & Hagger, 2006):

1. On a scale of 1–5 where 1 is strongly disagree and 5 is strongly agree, how strongly do you agree with the statement I intend to take part in screening for diabetes if it is offered to me in the next few years?
2. On a scale of 1–5 where 1 is very unlikely and 5 is very likely, how likely is it that you would take part in screening for diabetes if it were offered to you in the next few years?
3. On a scale of 1–5 where 1 is definitely do not and 5 is definitely do, how strongly do you agree with the statement I plan to take part in screening for diabetes if it were offered to me in the next few years?

*Socioeconomic status*. Housing tenure and educational qualifications were used as a proxy measure of SES. Participants were asked to indicate whether they had any formal educational qualifications and whether they owned their own house (including having a mortgage). Those who neither owned their houses nor had educational qualifications were considered to have the lowest SES (scored as 0), those who either owned their houses or had educational qualifications were considered to have intermediate levels of SES (scored as 1) and those who both owned their houses and had educational qualifications were considered to have the highest SES (scored as 2).

### Analyses

Discrimination was calculated by using the formula for Ferguson's delta (δ: Ferguson, 1949; Kline, 1993) modified for use with a polytomous outcome variable (δ_*G*_: Hankins, 2007). Coefficient delta is a non-parametric statistic and ranges between 0 (no discrimination) and 1 (maximum discrimination) with δ > 0.9 for a normal distribution of scores. Construct validity was initially explored using Spearman's rho correlations to identify the relationships between SES, time orientation and expectations of participation in screening. To further explore these relationships, mediation analyses were used to identify the indirect effect of time orientation on the relationship between SES and expectations of participation in diabetes screening using a macro developed for SPSS (Preacher & Hayes, 2004). This gives the separate effect sizes of the effect of the independent variable and mediator variable on the dependent variable and the Sobel value for the size of the indirect effect of the independent variable on the dependent variable. As the Sobel test assumes normal distribution, a bootstrap estimate for the indirect effect is given.

## Results

The associations between the variables of interest are shown in Table 3. ZTPI-R present showed a negative association with SES. There was a significant positive association between SES and future orientation as measured by the CFC but not as measured by the ZTPI-R (*p* = 0.247). In relation to expectations of participation in screening, there was a significant positive association with present orientation and a smaller positive association with future orientation as measured by the CFC but not as measured by the ZTPI-R. Both measures showed high discrimination (δ_*G*_ = 0.98 for the CFC and 0.97 for both sub-scales of the ZTPI-R).

**Table 3:** Associations between measures of time orientation, social deprivation and expectations of participation in diabetes screening (Spearman's rho correlations).

Psychological measures	CFC: future orientation	ZTPI-R future orientation	ZTPI-R present orientation	SES
Expectations of participation	0.18**	0.05	−0.27***	0.22***
CFC: future orientation		0.47***	−0.45***	0.14*
ZTPI future orientation			−0.30***	0.07
ZTPI present orientation				−0.25***

Notes:

* *p* < 0.05,

** *p* < 0.01 and

*** *p* < 0.001.

**Figure 2:**
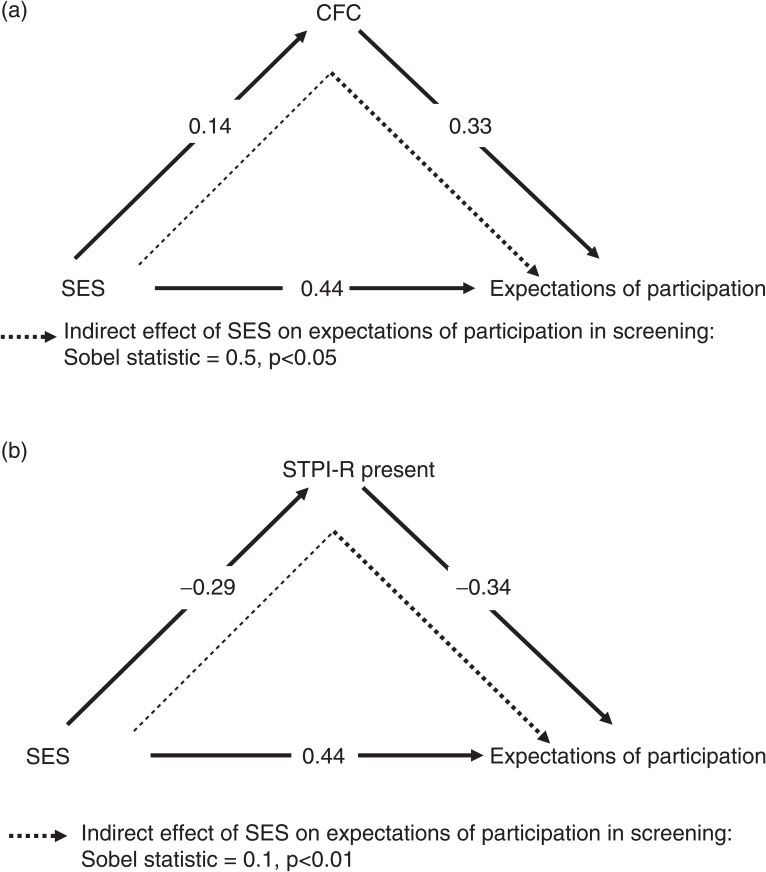
Relationships between SES, expectations of participation in diabetes screening, and (a) CFC future and (b) ZTPI-R present.

Because only the CFC future and ZTPI-R present orientation showed associations with SES and expectations of participation in screening, mediation analyses were conducted for association involving these measures only. Figure 2A illustrates the mediating effect of CFC future orientation on the relationship between SES and expectations of participation in screening. Controlling for social deprivation there were significant direct effects of SES on expectations of participation in screening (*b* = 0.44, *t* = 4.64, *p* < 0.001), of SES on CFC future orientation (*b* = 0.14, *t* = 2.87, *p* < 0.01) and of CFC future orientation on expectations of participation in screening (*b* = 0.33, *t* = 3.03, *p* < 0.01). There was also a significant direct effect of SES on expectations of participation in screening, controlling for CFC future orientation (*b* = 0.40, *t* = 4.14, *p* < 0.001). A Sobel test indicated that there was a significant indirect effect of SES on participation in screening mediated by CFC future orientation (Sobel value 0.05, 95%CI: 0.002–0.093, *p* < 0.05). This effect was bootstrapped, using 3000 re-samples, giving a mean effect of 0.05 (95%CI: 0.007–0.108). This result is similar and because zero is not in the 95% confidence intervals it can be presumed that the indirect effect is reliably different from zero. Figure 2B illustrates the mediating effect of ZTPI-R present orientation on the relationship between SES and expectations of participation in screening. There were significant direct effects of SES on present orientation (*b* = 0.29, *t* = 4.49, *p* < 0.001) and present orientation, controlling for SES, on expectations of participation in screening (*b* = 0.34, *t* = 4.03, *p* < 0.001). There was also a significant total direct effect of SES on expectations of screening, controlling for present orientation (*b* = 0.35, *t* = 3.59, *p* < 0.001). A Sobel test indicated a significant indirect effect of SES on participation in screening through present orientation (Sobel statistic 0.1, 95%CI: 0.033–0.162, *p* < 0.01). Bootstrapping with 3000 re-samples indicated a mean result for the indirect effect of 0.1 (95%CI: 0.039–0.171), similar to the result of the Sobel test.

## Discussion

Ferguson's δ indicated that all measures had good discrimination. Initial examination of the associations between time orientation, expectations of participation in screening and SES indicated that ZTPI-R present orientation was negatively associated with, and CFC future orientation more weakly and positively associated with, expectations of participation in screening and SES. ZTPI-R future was not significantly associated with expectations of participation in screening or SES. Both the CFC future orientation and ZTPI-R present orientation partially mediated the effects of social deprivation on expectations of participation although a stronger mediating pathway was indicated for the latter.

While the measures had good discrimination, a less clear picture emerged for their construct validity. Good construct validity was found for ZTPI-R present orientation which partially mediated the effect of SES on screening participation, but the results for future orientation as measured by the CFC and the ZTPI-R were contradictory. ZTPI-R future orientation did not mediate the relationship between SES and expectations of participation as it was not significantly associated with either variable. CFC future orientation partially mediated this relationship, although it was a weaker model than that provided by ZTPI-R present orientation.

These findings raise a number of issues. First, the current study lends support to the previously cited studies indicating that future and present orientation each account for unique variance in the prediction of health behaviour (Keough et al., 1999; Zimbardo et al., 1997) as the ZTPI-R future and present orientation sub-scales had different relationships with social deprivation and expectations of participation. Second, a question is raised by the finding that the ZTPI-R future orientation did not, as predicted, mediate the impact of social deprivation on expectations of participation in screening while CFC future orientation mediated this relationship, but more weakly than did ZTPI-R present orientation. One explanation for this weak effect is that the CFC artificially forces future and present orientation into polar opposites as 9 of the 12 items require participants to make an explicit choice between present and future outcomes. While the authors suggest that the CFC is measuring an aspect of future orientation, (Strathman et al., 1994) these results suggests that it is measuring a confound of present and future orientation, that is, a choice between the two time dimensions in evaluating the consequences of actions. Consequently, the mediating effect of the CFC cannot be held to be evidence that future orientation mediates the relationship between social deprivation and uptake of screening.

## General discussion and conclusions

Study 1 found that the reliability of the CFC was good but that the readability was poor. While the ZTPI-R is a more readable measure, its reliability has been compromised by the inclusion of items which do not measure time orientation. The results indicate that factor solutions involving factors comprised purely of reverse-scored items should be treated with caution. Further, the presence of reverse-scored items alongside non-reverse-scored items within a factor can have an impact on the measurement properties of a scale. While future orientation has been associated with various health-related behaviours, Study 2 suggests that it is present orientation which may be a stronger predictor of such behaviours and may therefore have greater potential in explaining why people do or do not engage in behaviours that could improve their health. All three measures had good discrimination.

The research presented in this article provides data on the properties of the CFC and the ZTPI which researchers can use to guide a choice of measure most appropriate to their own research. In the context of general population samples, with mixed levels of literacy, the ZTPI-R may be a better measure of time orientation to use given its greater readability. Additionally, the measurement by the ZTPI-R of both present and future orientation gives it particular utility in studies attempting to explain uptake of health behaviours with immediate harms and future benefits. Using the ZTPI-R as a basis, future work might focus on extending the number of items that measure future and present orientation in general populations without compromising the readability of these sub-scales. In addition, future work might consider the integration of time orientation into existing models of decision making in health and in particular Social Cognitive Theory (SCT). Central to SCT are the concepts of self-efficacy and outcome expectancies (Luszcznska & Schwarzer, 2005). Self-efficacy is associated with future orientation (Epel et al., 1999; Luszczynska et al., 2004) and outcome expectancies involve evaluation of both short- and long-term consequences of the decision (Luszcznska & Schwarzer, 2005) which this research suggests are likely to be influenced by the time orientation of the participant. Therefore, assessing the time orientation of participants might add to the explanatory power of this model.

The two studies reported here have some limitations. The data were cross-sectional so causation cannot be inferred. Nor is it possible to assess the extent to which time orientation may vary across situations, although the test-retest data provide some evidence that the concepts are stable over time. Finally, the use of analogue studies is a limitation as people may behave differently when actually invited to screening. But there is good reason to think that these results reflect how people behave when responding to an invitation to screening given that the social patterning of screening uptake in the current study reflects that found in studies reporting responses of those actually offered screening. (Kim et al., 2004; McCaffery et al., 2002; Sutton & Rutherford, 2005).

A number of conclusions can be drawn from the results of Studies 1 and 2. First, they indicate that measures designed to be used in general populations should be readable by those with the lowest levels of education and be validated with such groups. Second, further research is warranted on the interactive effects of reverse scoring of items, item readability and participants' education on the factor structure of questionnaires. Finally, these results provide evidence that engaging in health-related behaviours is related to present orientation. The findings raise the prospect of being able to tailor interventions to promote uptake of a health-related behaviour to the time orientation of participants. In particular, they suggest that targeting health promotion campaigns to those with low present orientation may help such campaigns reach low SES groups whose behaviour puts them at higher risk of ill health.

## Appendix 1. Participant information about diabetes screening

There are two types of diabetes. The most common type of diabetes is called Type 2 diabetes. People who have this type of diabetes are more likely to develop heart disease, stroke, eyesight problems, kidney failure, problems with feet and impotence and their life expectancy is reduced by 10 years. The complications of having diabetes can be prevented by controlling diet, increasing activity and by taking tablets prescribed by a doctor.

The chance of developing diabetes increases with age. It is possible for someone to have Type 2 diabetes and not to know it because they show no symptoms and do not feel unwell. The Department of Health estimates that there are between 600,000 and 800,000 people in this country who do not know that they have diabetes. The Department of Health is considering offering people a free screening test for diabetes in the next few years.

The screening test can find some but not all of those who have the early stages of diabetes. Some people find that taking part in screening means that they have to undergo unpleasant and inconvenient procedures. They may also find that they start worrying about diabetes and may have to start taking tablets and changing their lifestyle. Some people find that taking part in screening gives them peace of mind about their health and they also know that their early diagnosis will prevent complications and illness.

## Appendix 2. The Consideration of Future Consequences Scale

Readability scores shown by GFI.

1. I consider how things might be in the future, and try to influence those things with my day-to-day behaviour (GF1 = 12.21).
2. Often I engage in a particular behaviour in order to achieve outcomes that may not result for many years (GFI = 11.81).
3. I only act to satisfy immediate concerns; figuring the future will take care of itself (GFI = 14.00).^a^
4. My behaviour is only influenced by the immediate (i.e., a matter of days or weeks) outcomes of my actions (GFI = 14.17).^a^
5. My convenience is a big factor in the decisions I make or the actions I take (GFI = 11.40).^a^
6. I am willing to sacrifice my immediate happiness or well-being in order to achieve future outcomes (GFI = 13.90).
7. I think it is important to take warnings about negative outcomes seriously even if the negative outcome will not occur for many years (GFI = 16.16).
8. I think it is more important to perform a behaviour with important distant consequences than a behaviour with less-important immediate consequences (GFI = 23.64).
9. I generally ignore warnings about possible future problems because I think the problems will be resolved before they reach crisis level (GFI = 12.21).^a^
10. I think that sacrificing now is usually unnecessary since future outcomes can be dealt with at a later time (GFI = 13.92).^a^
11. I only act to satisfy immediate concerns; figuring that I will take care of future problems that may occur at a later date (GFI = 14.42).^a^
12. Since my day-to-day work has specific outcomes, it is more important to me than behaviour that has distant outcomes (GFI = 16.02).^a^

a Reverse-scored items.

## Appendix 3. The Zimbardo Time Perspective Inventory (22 item version)

Readability scores shown by GFI.

1. I believe that a person's day should be planned ahead each morning (GFI = 5.20).^a^
2. Thinking about the future is pleasant to me (GFI = 3.20).^b^,^c^
3. When I want to achieve something I set goals and consider specific means of reaching those goals (GFI = 13.86).^b^,^c^
4. Meeting tomorrow's deadlines and doing other necessary work comes before tonight's play (GFI = 11.31).^b^,^c^
5. It seems to me that my future plans are pretty well laid out (GFI = 5.29).^b^,^c^
6. I think that it seems useless to plan too far ahead because things hardly ever come out the way you planned anyway (GFI = 10.62).^a^,^c^&^d^
7. It upsets me to be late for appointments (GFI = 8.20).^e^
8. I tend to loose my temper when I am provoked (GFI = 8.00).^a^
9. I get irritated at people who keep me waiting when we have agreed to meet at a specific time (GFI = 11.81).^a^
10. I complete projects on time by making steady progress (GFI = 3.60).^e^
11. I make lists of things to do (GFI = 2.80).^a^
12. I keep working at a difficult uninteresting task if it will help me to get ahead (GFI = 11.40).^e^
13. I am able to resist temptations when I know that there is work to be done (GFI = 8.90).^e^
14. I do things impulsively, making decisions on the spur of the moment (GFI = 11.47).^f^
15. I believe that getting together with friends is one of life's important pleasures (GFI = 11.31).^f^
16. If I do not get done on time, I do not worry about it (GFI = 5.60).^a^,^h^
17. I try to live one day at a time (GFI = 3.60).^g^,^h^
18. Its fun to gamble when I have some extra money (GFI = 4.40).^f^
19. I feel that it is more important to enjoy what you are doing than to get the work done on time (10.30).^g^,^h^
20. I do not do things that are good for me if they do not feel good now (6.80).^g^,^h^
21. I get drunk at parties (GFI = 2.00).^f^
22. I take risks to put excitement into my life (GFI = 8.04).^f^

a Items loading on to factor not included in final solution. b Items loading on future factor. c Items comprising ZTPI-R future. d Reverse scored in short version of the ZTPI-R. e Items loading on conscientiousness factor. f Items loading on hedonism factor. g Items loading on to present factor. h Items comprising ZTPI-R present.
